# Compatibility of the evolution equation for heat flux in dual-phase-lag and three-phase-lag with the principles of thermodynamics

**DOI:** 10.1038/s41598-025-33764-3

**Published:** 2026-01-20

**Authors:** Asmaa Fawzy, W. Mahmoud, E. K. Rawy, A. F. Ghaleb

**Affiliations:** Department of Mathematics, Faculty of Science, Cairo University, Giza, 12613 Egypt

**Keywords:** Materials science, Mathematics and computing, Physics

## Abstract

In this work, we investigate the compatibility with the second law of thermodynamics of certain evolution equations for heat flux which frequently appear in the literature when treating problems of extended thermodynamics, namely in dual-phase-lag (DPL) and in three-phase-lag (TPL) theories for a rigid thermal conductor. For each one of these two cases, we propose a concrete form for the free energy function, in which heat flux enters as an independent variable side by side with temperature, and a corresponding non-negative quadratic dissipation function. An important aspect of the present work is that all the introduced material tensors in the formulation of the free energy are kept to the simplest form possible, and can be calculated based on experimental data. The present results demonstrate that both DPL and TPL approximate models for the evolution equation of the heat flux considered here can be rendered thermodynamically admissible through a class of free energy functions to satisfy the Second Law, providing a flexible and consistent modeling of non-Fourier heat conduction. In any case, it is shown that temperature and heat flux are determined simultaneously from a set of coupled, nonlinear partial differential equations, and that obtaining an equation for temperature independently from heat flux could be achieved only in special cases and under some simplifying assumptions. Within this context, the heat conductivity is assumed a linear function of temperature, an essential feature from an experimental point of view. The used methodology, consisting of going from evolution equations for the heat flux to the construction of dissipation functions and free energies which satisfy the requirements of the second law, is, in our belief, a useful trend to treat more difficult cases for higher approximation theories, or for couplings with the other fields, i.e. dynamics, electromagnetism and other. In spite of slight resemblance with published work, the suggested concrete forms of the free energies and dissipation functions, as well as the governing nonlinear set of partial differential equations are not mentioned in the available literature to the authors knowledge. It turns out that the only requirement of consistency with the second law of thermodynamics for the treated cases is that the thermal relaxation times be non-negative, and that the thermal relaxation time related to thermal displacement in TPL satisfies a certain inequality. Any further requirements could be linked only to the stability of solutions of the arising governing partial differential equations. A one-dimensional application in a half-space solves the newly suggested nonlinear system of governing equations, giving a physical insight to the presented model, and pointing out at its adequacy for describing the propagation of thermal waves with finite speed.

## Introduction

Extended Thermodynamics englobes several thermodynamical theories for the description of heat conduction, differing among themselves in formalism, physical insight, relation to experiment, domain of validity and domain of applicability. Extended Irreversible Thermodynamics (EIT), Rational Extended Thermodynamics (RET), Conservation-Dissipation Formalism (CDF) and General Equation for Non-Equilibrium Reversible-Irreversible Coupling (GENERIC) are examples of such theories. Most of the thermodynamical theories requires a definite set of state variables, in terms of which the different evolution laws are to be formulated. For GENERIC, however, one remains “uncommitted” to the choice of state variables (C.f.^1^). A large amount of literature has been devoted to each of these theories, and to the comparison between them, among which one cites^1,2^ for the sake of conciseness.

Within the EIT theory presently considered, the heat flux plays a central role as an additional thermodynamical state variable to be included in the relevant thermodynamical functions, together with temperature. In the presented work, we try to reach thermodynamical compatibility of the evolution equation for heat flux in two models DPL and TPL of heat conduction. Nonlinearity up to the second order is included, mainly through the dependence of thermal conductivity on temperature, a fact that may be of practical interest in the modeling of media with complex structure.

The relation between heat flux and temperature gradient is given by the constitutive relation of heat flux which, along with the First Law of Thermodynamics or the energy conservation equation, yields the heat conduction equation. The first mathematical description of this relation was proposed by Fourier in 1822 , that is,

1 $$\begin{aligned} \boldsymbol{Q}=- K \boldsymbol{\nabla } \theta , \end{aligned}$$

which is widely applied in engineering at present. Here, $\boldsymbol{Q}$ is the heat flux vector, $\theta$ is the temperature as measured from a reference temperature $\Theta _0$, and *K* is a positive scalar named the coefficient of heat conduction. However, Fourier’s law predicts that the heat waves propagate with infinite speed. This means that thermal disturbances are felt instantaneously at all points far from the thermal source, which is physically unrealistic for applications involving very low temperatures, ultrafast laser heating, spatial microscales and micromechanics. As a consequence, different non-Fourier constitutive relations and heat conduction equations have been introduced to remove this paradox of the classical Fourier heat conduction. The constitutive heat flux relation with hyperbolic-type heat conduction equation was proposed independently by Cattaneo^3^ and Vernotte^4^, based on the following modification of Fourier’s law for heat conduction:

2 $$\begin{aligned} \tau _q \frac{\partial \boldsymbol{Q}}{\partial t} + \boldsymbol{Q}=- K \boldsymbol{\nabla } \theta (x,t). \end{aligned}$$

In this model, the time-lag needed for the establishment of heat flux was characterized by the thermal relaxation time $\tau _q$. Relation (2) leads to a hyperbolic-type heat conduction and predicts the propagation of thermal waves with finite speed, called second sound. These waves have observed, for example, in liquid helium 3, a phenomenon large enough to yield measurable wave effects. Some of the predictions of this model are not compatible with the experimental results.

A large amount of literature was devoted to the investigation of the model of heat conduction with finite wave speed and one thermal relaxation time. A novel theory of heat conduction with finite wave speed was proposed by Gurtin and Pipkin^5^. In their theory, the heat flux is obtained from the free energy of the system, not as a kinetic equation. Following a different approach, Coleman et al.^6^ found that the set of arguments of the free energy must be enriched in this case by adding the heat flux, side by side with temperature, in order to correctly describe the propagation of heat as wave. Explanations were given for such an addition. Along the same guidelines, Ghaleb^7^ investigated a model of thermoelasticity, Bai and Lavine^8^ introduced a modified hyperbolic type heat conduction equation which is consistent with the second law of thermodynamics, with a one-dimensional example of heat propagation in a finite interval. Abou-Dina et al.^9^ considered electro-thermoelasticity, while Ahmed et al.^10^ investigated a two-dimensional model of thermoelasticity, both with one relaxation time.

A further step was undertaken with the aim of evaluating microstructural effects such as phonon-electron interaction and phonon scattering. The heat flux constitutive relation proposed by Tzou^11,12^ considers the phase-lag of temperature gradient along with the phase-lag of heat flux. The arising DPL model includes two thermal relaxation times. The relaxation time $\tau _{\theta }$ is due to microstructural interactions such as phonon scattering or phonon-electron interactions, while the relaxation time $\tau _q$ is caused by fast-transient effects of thermal inertia. Later on, this model became key to understanding heat transfer in nanomaterials, plasma, and biological systems. Ván^13,14^, Ván and Füllöp^15^. Kovács and Ván^16^ derived a novel equation of heat conduction using a generalized entropy current and internal variables, developed a numerical solution scheme and proved its stability. The same authors^17^ confirmed the introduction of heat flux in the free energy in DPL to investigate weakly nonlocal irreversible thermodynamics based on generalized entropy current, nonlinearities and concrete forms of the dissipation function but no concrete forms for the free energy, with several examples of heat conduction equations.

Different approximations of the evolution law for heat flux in dual-phase-lag exist in the literature (C.f.^17–20^). Correspondingly, there is a multitude of heat conduction partial differential equations of different orders describing finite speed heat propagation (C.f.^17,18^).

In the early 2000’s, the TPL model was developed as an extension of dual-phase-lag. Starting from the Green–Naghdi model, Choudhuri^21^ introduces the phase-lag of heat flux, temperature gradient, and thermal displacement gradient in the heat conduction equation. These phase lags represent the finite time needed for thermal disturbances to propagate, thus enabling a more accurate modeling of microscale and transient thermal phenomena.

The existence of an evolution equation for the heat flux clearly means that heat flux and temperature must now be treated as independent thermodynamical variables. A consequence of this is that the dissipation function is not necessarily limited to a product of heat flux and temperature gradient, while the expressions for entropy acquire additional quadratic terms in the heat flux (C.f.^13–15,17^).

An extensive literature was devoted to the investigation of concrete problems related to the DPL and TPL theories (C.f.^22–24^). In particular, some anomalous negative temperatures appearing during heating were reported concerning DPL (C.f.^25,26^). However, such an anomaly could be attributed to non-realistic values assigned to the thermal relaxation times (C.f.^27^), or could be removed by proper adjustment of the model (C.f.^8^).

A detailed account of the extension of the domain of application of thermodynamics to nanosystems may be found in the book by Sellito, Cimmelli and Jou^28^. Munafò et al.^29^ proposed a two-dimensional nonlinear hyperbolic heat transport equation based on the Cattaneo model for a rigid thermal conductor, with thermal conductivity and relaxation time linearly dependent on temperature. Kovács^30^ reviews models from the Rational Extended Thermodynamics, Extended Irreversible Thermodynamics, and Non-Equilibrium Thermodynamics with internal variables frameworks and discusses their origins, limitations, stability conditions, and the phenomena to which they can be applied. Recently, Giorgi et al.^31^ derived rate-type equations for the description of the behavior of heat flow in deformable media. They presented free energy and the entropy production forms in terms of a set of thermodynamical variables that comply with the requirements of the Second Law. Their analysis was shown to provide already known models, such as the Cattaneo-Vernotte and the Jeffrey-like models for thermal conductors. The influence of the temperature dependence of thermal conductivity and thermal relaxation time is investigated by Munafò et al.^32^ within two generalizations of the Maxwell-Cattaneo-Vernotte model, with numerical solutions. Heat transfer at nanoscale and the wall contributions to the local heat flux is studied by Munafò et al.^33^ and by Bochicchio et al.^34^.

The overwhelming majority of the existing literature on DPL and TPL is limited to the solution of the linear heat conduction equation for temperature under different settings, while only very few consider the complete model of heat conduction and deals with the mathematical problems of existence, uniqueness and stability of solutions, and the well-posedness of the problem in general.

Following the guidelines expressed in^6^ for the purely thermal case and^9^ for thermoelastic media in assuming that the free energy of the complex system depends on the heat flux vector, alongside with temperature within the framework of extended thermodynamics, the purpose of the present work is as follows: Starting with a well-known established form of the evolution equation for the heat flux in DPL or TPL, to propose corresponding concrete forms for the free energy function, the dissipation function and the entropy of the purely thermal system, that ensure thermodynamical consistency with the second law of thermodynamics in dual-phase-lag and in three-phase-lag theories, i.e produce a non-negative dissipation function in each case. During this process, all introduced material tensors are kept simplest in their expressions and, moreover, are related measurable quantities. The thermal conductivity is taken to depend linearly on temperature. The proposed forms for the free energy differ from the classical one by quadratic terms in the heat flux, temperature gradient and thermal displacement gradient components, thus leading to generalized expressions for the heat capacity that can be compared with possible experimental data. Again, the dissipation function is not necessarily limited to its known classical form as a scalar product of heat flux and temperature gradient, while the entropy acquires additional terms. The proposed model leads finally to a novel system of governing nonlinear partial differential equations which may be solved for the simultaneous determination of temperature and heat flux in DPL, in addition to the thermal displacement function in TPL. A simplified version of these equations, when only quadratic nonlinearities are retained, is mentioned for application. A one-dimensional example on DPL in a half-space allows to illustrate the reliability of the proposed model through 2D and 3D plots for the behavior of the solution, and to give a physical insight that points out at its adequacy in describing the propagation of finite speed heat waves in a rigid thermal conductor, and in producing acceptable solutions.

##  Evolution equations for the heat flux in dual- and three-phase-lag theories

In modeling non-Fourier heat conduction, such as DPL and TPL, the classical Fourier law is modified to include time delays in the response of matter to the propagation of heat. These delays are known to represent physical processes, like microsrtucture interaction and finite thermal wave speed that cannot be captured by the classical theory. The following equations, considered as efficient approximations that allow to describe how the heat flux evolves with time in DPL and TPL, are frequently met in the literature. They will serve as the main ingredients for the current analysis.

1. Evolution equation in dual-phase-lag theory:
  3 $$\begin{aligned} \boldsymbol{Q}+\tau _q \dot{\boldsymbol{Q}} =- \boldsymbol{K} \left( \boldsymbol{\nabla } \theta +\tau _{\theta }\frac{\partial \boldsymbol{\nabla } {\theta }}{\partial t}\right) , \end{aligned}$$
  with:    $\boldsymbol{Q}$    Vector of heat flux
     $\theta$     Temperature measured from a reference temperature $\Theta _0, \Theta = \Theta _0 + \theta$
     $\tau _{q}$     Phase lag of the heat flux, may be temperature dependent
     $\tau _{\theta }$     Phase lag of the temperature gradient, may be temperature dependent
     $\boldsymbol{K}$     Tensor of thermal conductivity, taken symmetric and positive definite, may be temperature dependent
2. Evolution equation in three-phase-lag forms:
  4 $$\begin{aligned} \boldsymbol{Q}+\tau _q \dot{\boldsymbol{Q}}=- \boldsymbol{K} (\boldsymbol{\nabla } \theta +\tau _{\theta } \ \boldsymbol{\nabla }{\dot{\theta }})- \boldsymbol{K}^* (\boldsymbol{\nabla }{\upsilon }+\tau _{{\upsilon }} \boldsymbol{\nabla } {\dot{{\upsilon }}} ) \end{aligned}$$
  or, equivalently
  5 $$\begin{aligned} \boldsymbol{Q}+\tau _q\dot{\boldsymbol{Q}}=- ( \boldsymbol{\kappa } \boldsymbol{\nabla } \theta + \tau _{\theta } \boldsymbol{K} \boldsymbol{\nabla }{\dot{\theta }}+ \boldsymbol{K}^* \boldsymbol{\nabla }{\upsilon } ), \end{aligned}$$
  where
  6 $$\begin{aligned} \boldsymbol{\kappa } = \boldsymbol{K} +\tau _{\upsilon }\boldsymbol{ K}^* , \qquad \dot{\upsilon } =\theta , \end{aligned}$$
  with:    $\upsilon$    Thermal displacement
     $\tau _{\textit{v}}$    Phase lag of thermal displacement
     $\boldsymbol{K}^*$ Thermal conductivity rate tensor, taken symmetric and commuting with $\boldsymbol{K}$

## Dual-phase-lag theory

The consistency of the dual-phase-lag heat conduction equation with the second law of thermodynamics, and the qualitative properties of the solution were investigated in^13–15,17,19,20^. Consistency in some of these references is understood in the sense that the integration of the product of heat flux and temperature gradient over thermodynamical cycles must be non-positive, which is the case for classical thermodynamics. For others, consistency means that the (quadratic) dissipation function must be non-negative. The form of the free energy of the system, and the arguments upon which it depends, do not intervene in the considerations. This is different from the present approach, where concrete free energy and dissipation function are defined in terms of some generalized arguments, then the non-negativity of the dissipation function is verified. It is believed that such an approach is advantageous, as it produces specific forms for the free enegy and the dissipation function in terms of an enriched set of arguments and, moreover, can be applied, with proper changes, to the three-phase lag heat conduction equation and to more elaborate models.

### Free energy function for dual-phase-lag

The authors are not aware of any concrete forms of the free energy in the investigations concerning DPL and TPL. Note that Giorgi et al. have produced expressions for the free energy for models of heat conduction through rate equations, including Jeffrey-type model that is parent to DPL. The free energy function is chosen along the guidelines that it be a reasonably simple quadratic form in the thermodynamical state variables. For the DPL model under consideration, it is taken in the form:

7 $$\begin{aligned} \psi =\psi _0 \left( \varepsilon _{ij},\theta \right) +\frac{1}{2} N_{ij}Q_{i}Q_{j}+M_{ij} Q_i \theta _{,j}+\frac{1}{2} P_{ij} \theta _{,i }\theta _{,j}, \end{aligned}$$

where $\psi _0$ is the energy function for a thermoelastic material in classical thermodynamics, $\varepsilon _{ij}$ is the infinitesimal strain tensor. Tensors $\boldsymbol{N}, \boldsymbol{M}$ and $\boldsymbol{P}$ are all assumed symmetric and dependent on the temperature $\theta$. The comma denotes covariant derivative and the dot, the material time derivative, as defined in^9^. It is worth noting here that the quadratic dependence of the free energy on heat flux was derived by Coleman et al.^6^ from thermodynamical considerations for the case of a single thermal relaxation time. Moreover, it was shown that the free energy of the system in that case cannot include the gradient of temperature among its arguments.

### Clausius–Duhem inequality and dissipation function for dual-phase-lag

Following^9^, the constitutive equation for stress depends only on the function $\psi _0$ and, therefore, has the same form as in classical thermoelasticity, while the entropy acquires an additional quadratic expression involving the heat flux and the temperature gradient:

8 $$\begin{aligned} p_{ij}= & \rho \frac{\partial \psi }{\partial \varepsilon _{ij}} = \rho \frac{\partial \psi _0}{\partial \varepsilon _{ij}} = p_{ij}^0, \end{aligned}$$

9 $$\begin{aligned} \eta= & - \frac{\partial \psi }{\partial \theta } = \eta _0 - \frac{1}{2} N'_{ij}Q_{i}Q_{j}- \frac{1}{2} M'_{ij} \left( Q_i \theta _{,j} + Q_j \theta _{,i} \right) - \frac{1}{2} P'_{ij} \theta _{,i }\theta _{,j}, \end{aligned}$$

where the ‘dash’ denotes differentiation w.r.to $\theta$ and $\eta _0 =- \frac{\partial \psi _0}{\partial \theta }$ is the expression for the entropy in classical thermodynamics. It is thus seen that the expression for the entropy density function acquires additive terms as compared to the classical case, all depending on the temperature dependence of the thermal conductivity.

We shall restrict further considerations to the rigid thermal conductor. Expression (9) may be compared to the one shown in^17^, in which the addition to the classical expression for entropy consists of a quadratic term in an internal parameter, which is often taken to be the heat flux, but could have a more general expression, so it can generate (9) if this internal parameter is taken as a combination of the heat flux and temperature gradient.

The remaining part of the energy equation, in conjunction with the second law of thermodynamics, reduces to the well-known Clausius-Duhem inequality for the dissipation function:

10 $$\begin{aligned} \sigma = -\rho \frac{\partial \psi }{\partial Q_i}\dot{Q_i}-\rho \frac{\partial \psi }{\partial \theta _{,i}}\dot{\theta _{,i}}-\frac{Q_i}{\Theta }\theta _{,i} \ge 0 \end{aligned}$$

Having in mind to restrict our considerations to quadratic nonlinearities included, we shall replace $\Theta$ by $\Theta _o$ as an approximation in the above equation. Now substitute for the heat flow rate from (3) in (10) to get:

$$\begin{aligned} \tau _q \sigma = -\rho \frac{\partial \psi }{\partial Q_i} \left[ -Q_i - K_{ij}(\theta _{,j} +\tau _{\theta }\dot{\theta _{,j}}) \right] -\rho \tau _q \frac{\partial \psi }{\partial \theta _{,i}}\dot{\theta _{,i}}- \tau _q \frac{Q_i}{\Theta _o}\theta _{,i}. \end{aligned}$$

Simplify this last equation using expression (7) for the free energy to finally get:

$$\begin{aligned} \begin{aligned} \tau _q\sigma&= \rho N_{ij}Q_iQ_j+\rho M_{ji}\theta _{,i}Q_j+\rho N_{ij}Q_j K_{ik} \theta _{,k}+\rho M_{ij}\theta _{,j} K_{ik} \theta _{,k} + \rho N_{ij}Q_j \tau _{\theta } K_{ik} \dot{\theta _{,k}} \\&\quad + \rho M_{ij}\theta _{,j} K_{ik} \tau _{\theta } \dot{\theta _{,k}}-\rho \tau _q M_{ij}Q_i \dot{\theta _{,j}} -\rho \tau _qP_{ij}\theta _{,j} \dot{\theta _{,i}} -\tau _q\frac{Q_i}{\Theta _o}\theta _{,i}. \end{aligned} \end{aligned}$$

Choosing simple special expressions for the different material tensors figuring in the matrix associated with the dissipation function, allows to reduce this matrix to a form of blocks along the main diagonal:

$$\begin{aligned} \boldsymbol{M}= & \frac{\tau _q \tau _{\theta }}{\rho \Theta _o(\tau _{q} + \tau _{\theta })} \boldsymbol{I}_3, \\ \boldsymbol{P}= & \frac{\tau _{\theta }^2}{\rho (\tau _{q} + \tau _{\theta })\Theta _o} \boldsymbol{K}, \\ \boldsymbol{N}= & \frac{\tau _{q}^2}{\rho (\tau _{q} + \tau _{\theta })\Theta _o} \boldsymbol{K}^{-1}. \end{aligned}$$

Tensors $\boldsymbol{N}, \boldsymbol{M}$ and $\boldsymbol{P}$ are expressed in terms of two thermal relaxation times and the tensor of thermal conductivity, by which they acquire a physical meaning, and which makes them easy to calculate based on experimental data. Moreover, their dependence on temperature is clear from definition. Accodingly, the free energy and the dissipation function take on the final simplified forms in tensorial notation:

11 $$\begin{aligned} \psi= & \psi _0 \left( \varepsilon _{ij},\theta \right) +\frac{1}{2} \frac{\tau _{q}^2}{\rho (\tau _{q} + \tau _{\theta })\Theta _o} \boldsymbol{Q.} \boldsymbol{K^{-1}} \boldsymbol{Q} + \frac{\tau _q \tau _{\theta }}{\rho \Theta _o(\tau _{q} + \tau _{\theta })} \boldsymbol{Q.} \boldsymbol{\nabla } \theta +\frac{1}{2} \frac{\tau _{\theta }^2}{\rho (\tau _{q} + \tau _{\theta })\Theta _o} \boldsymbol{\nabla } \theta \boldsymbol{. K} \boldsymbol{\nabla } \theta , \end{aligned}$$

12 $$\begin{aligned} \sigma= & \frac{\tau _{q}^2}{(\tau _{q} + \tau _{\theta })\Theta _o} \boldsymbol{Q.} \boldsymbol{K^{-1}} \boldsymbol{Q} + \frac{\tau _{q} \tau _{\theta }}{(\tau _{q} + \tau _{\theta })\Theta _o} \boldsymbol{\nabla } \theta \boldsymbol{. K} \boldsymbol{\nabla } \theta . \end{aligned}$$

Such a concise form of the dissipation function where the thermal conductivity tensor figures is not found in the literature. It is a direct generalization of the one given in^6^ for the case of a single thermal relaxation time. Parent relations to (11) and (12) may be found in^31^ for Jeffrey-type model.

The expression for the dissipation function $\sigma$ given in (12) is novel. It has two types of arguments: $Q_i,\theta _{, i}$ and all these are assumed bounded. Hence the vectors above are all bounded. Now take tensor $\boldsymbol{K}$ to be positive definite, so that $\boldsymbol{a}^T K \boldsymbol{a} > 0$ for any nonzero vector $\boldsymbol{a}$. It then follows that $\boldsymbol{K}^{-1}$ is symmetric and positive definite as well. Hence $\sigma \ge 0$. Note that the present form of the dissipation function is different from the well-known form in classical thermodynamics, and reflects the independence of heat flux and temperature as thermodynamical variables.

It may be noticed that for the case of a single thermal relaxation time $\tau _q$ (set $\tau _{\theta }=0$), the term containing the temperature gradient completely disappears from the dissipation function, and as an argument in the free energy, in agreement with the previous result in^6^.

In order to relate the introduced material tensors $\boldsymbol{M}$ and $\boldsymbol{P}$ to measurable quantities, let us calculate the heat capacity per unit volume from the well-known relation

13 $$\begin{aligned} c = \Theta \frac{\partial \eta }{\partial \theta }. \end{aligned}$$

One obtains:

14 $$\begin{aligned} c = c_0 - \frac{1}{2} N''_{ij} \, \Theta \, Q_{i}Q_{j}- \frac{1}{2} M''_{ij} \, \Theta \left( Q_i \theta _{,j} + Q_j \theta _{,i} \right) - \frac{1}{2} P''_{ij} \, \Theta \, \theta _{,i }\theta _{,j}, \end{aligned}$$

where $c_0$ is the heat capacity at equilibrium. In case the second derivatives $N'', M'', P''$ all vanish, the heat capacity keeps its value $c_0$ unchanged. In particular, this will be the case when the thermal relaxation times and the tensor of thermal conductivity are all independent of temperature.

## Three-phase-lag theory

The three-phase-lag theory of heat conduction was investigated by Chirita et al.^35,36^ for consistency with the basic laws of thermodynamics, and for some properties of the solution along the same guidelines as stated earlier for the dual-phase-lag.

### Free energy function for three-phase-lag

The free energy function is taken in the form:

15 $$\begin{aligned} \psi= & \psi _0 \left( \varepsilon _{ij},\theta \right) +\frac{1}{2} N_{ij}Q_{i}Q_{j}+M_{ij} Q_i \theta _{,j}+\frac{1}{2} P_{ij} \theta _{,i }\theta _{,j} +\mu _{ij}Q_i \upsilon _{,j} \nonumber \\ & \quad + \beta _{ij} \theta _{,i} \upsilon _{,j}+\frac{1}{2} \alpha _{ij} \upsilon _{,i} \upsilon _{,j}, \end{aligned}$$

where $\psi _0$ has been defined earlier. Tensors $\boldsymbol{N}, \boldsymbol{P}$ and $\boldsymbol{\alpha }$ are all taken symmetric, while $\boldsymbol{\mu }$ is assumed skew-symmetric.

### Clausius–Duhem inequality and dissipation function for three-phase-lag

The constitutive relations for stress and entropy are the same as in (8). The remaining part of the energy equation reduces to Clausius-Duhem inequality for the dissipation function:

16 $$\begin{aligned} \sigma = -\rho \frac{\partial \psi }{\partial Q_i}\dot{Q_i}-\rho \frac{\partial \psi }{\partial \theta _{,i}}\dot{\theta _{,i}}-\rho \frac{\partial \psi }{\partial \upsilon _{,i}}\dot{\upsilon _{,i}}-\frac{Q_i}{\Theta _o}\theta _{,i} \ge 0. \end{aligned}$$

Now substitute by (5) and the second of (6) in (16) to get:

17 $$\begin{aligned} \tau _q\sigma= & -\rho ( N_{ij}Q_j +M_{ij}\theta _{,j}+\mu _{ij} \upsilon _{,j}) \times \nonumber \\ & \times (-Q_i-\kappa _{ik} \theta _{,k} -K_{ik}\tau _{\theta } \dot{\theta }_ {,k}-K_{ik}^*\upsilon _{,k})- \nonumber \\ & \rho \tau _q( M_{ji}Q_j +P_{ij}\theta _{,j} +\beta _{ij}\upsilon _{,j})\dot{\theta _{,i}}- \nonumber \\ & \rho \tau _q( \mu _{ji}Q_j +\beta _{ji}\theta _{,j}+\alpha _{ij}\upsilon _{,j})\theta _{,i} -\tau _q\frac{Q_i}{\Theta _o}\theta _{,i} \end{aligned}$$

The following choices of the different quantities

18 $$\begin{aligned} & \boldsymbol{N} \left[ \left( \tau _{\theta } + \tau _q \right) \boldsymbol{K} + \tau _q \left( \tau _{\upsilon } + \frac{1}{2} \tau _q \right) \boldsymbol{K}^* \right] - \frac{1}{2} \tau _q^2 \, \boldsymbol{K}^* \boldsymbol{N} - \frac{\tau _q^2}{\rho \Theta _o} \boldsymbol{I}_3 = 0, \end{aligned}$$

19 $$\begin{aligned} & \boldsymbol{\mu } = \frac{1}{2} \left( \boldsymbol{N} \boldsymbol{K}^* - \boldsymbol{K}^* \boldsymbol{N} \right) , \end{aligned}$$

20 $$\begin{aligned} & \tau _q \boldsymbol{\beta } = - \frac{1}{2} \tau _{\theta } \boldsymbol{K} \left( \boldsymbol{N} \boldsymbol{K}^* - \boldsymbol{K}^* \boldsymbol{N} \right) , \end{aligned}$$

21 $$\begin{aligned} & \tau _q \boldsymbol{M} = \tau _{\theta } \boldsymbol{N} \boldsymbol{K}, \end{aligned}$$

22 $$\begin{aligned} & \tau _q \boldsymbol{P} = \tau _{\theta } \boldsymbol{K} \boldsymbol{M}, \end{aligned}$$

23 $$\begin{aligned} & \tau _q^2 \boldsymbol{\alpha } = - \frac{1}{2} \tau _q \left( \boldsymbol{K} + \tau _{\upsilon } \boldsymbol{K}^* \right) \left( \boldsymbol{N} \boldsymbol{K}^* - \boldsymbol{K}^* \boldsymbol{N} \right) + \tau _{\theta } \boldsymbol{N} \boldsymbol{K} \boldsymbol{K}^* \end{aligned}$$

reduce the dissipation function to the simple form (free of the variables $\upsilon _{,i}$):

24 $$\begin{aligned} \tau _q \sigma = \rho N_{ij}Q_iQ_j +\rho G_{ij} \theta _{,i} \, \theta _{,j}, \end{aligned}$$

with

25 $$\begin{aligned} G_{ij} = M_{ki} \kappa _{kj} - \tau _q \beta _{ij}. \end{aligned}$$

Thus:

26 $$\begin{aligned} \boldsymbol{G} = \frac{\tau _{\theta }}{\tau _q} \boldsymbol{K} \boldsymbol{N} \left[ \boldsymbol{K} + \left( \tau _{\upsilon } + \frac{1}{2} \tau _q \right) \boldsymbol{K}^* \right] - \frac{1}{2} \tau _{\theta } \boldsymbol{K} \boldsymbol{K}^* \boldsymbol{N} \end{aligned}$$

is expressed in terms of the three thermal relaxation times, and two tensors of thermal conductivity $\boldsymbol{K}$ and $\boldsymbol{K}^*$. All these quantities acquire a physical meaning, and can be measured experimentally.

The dissipation function is now:

27 $$\begin{aligned} \tau _q \sigma = \rho N_{ij}Q_iQ_j +\rho G_{ij} \theta _{,i} \, \theta _{,j}. \end{aligned}$$

It has two types of arguments: $Q_i$ and $\theta _{, i}$ as in DPL, and does not include the thermal displacement.

The algebraic equation (18) for $\boldsymbol{N}$ can be easily solved if it is transferred to the principal axes of the positive definite tensor $\boldsymbol{K}$. In fact, let us perform a simultaneous diagonalization of the two commuting tensors $\boldsymbol{K}$ and $\boldsymbol{K}^*$ by means of the orthogonal transformation *S*, say:

28 $$\begin{aligned} S^T \boldsymbol{K} S = \boldsymbol{I}_3, \qquad S^T \boldsymbol{K}^* S = \boldsymbol{D}^* = \text {diag} \{d_1^*, d_2^*, d_3^* \}. \end{aligned}$$

If $\bar{\boldsymbol{N}} = \left( \bar{N}_{ij} \right)$ denotes the tensor $S^T \boldsymbol{N} S$, then Eq. (18) yields:

29 $$\begin{aligned} \bar{\boldsymbol{N}} \left( \left( \tau _q + \tau _{\theta } \right) \boldsymbol{I}_3 + \tau _q \left( \tau _{\upsilon } + \frac{1}{2} \tau _q \right) \boldsymbol{D}^* \right) - \frac{1}{2} \tau _q^2 \, \boldsymbol{D}^* \bar{N} = \frac{\tau _q^2}{\rho \Theta _o} \boldsymbol{I}_3. \end{aligned}$$

Equating the entry (*i*, *j*) on both sides of this last equation yields:

30 $$\begin{aligned} & \bar{N}_{ij} \left( \left( \tau _q + \tau _{\theta } \right) + \tau _q \left( \tau _{\upsilon } + \frac{1}{2} \tau _q \right) d_j^* \right) -\frac{1}{2} \tau _q^2 \, d_i^* \bar{N}_{ij} \nonumber \\ & \quad = \frac{\tau _q^2}{\rho \Theta _o} \delta _{ij}. \quad \text {no summation over indices!} \end{aligned}$$

When $$i \ne j$$:

31 $$\begin{aligned} \bar{N}_{ij} \left( \left( \tau _q + \tau _{\theta } \right) + \tau _q \left( \tau _{\upsilon } + \frac{1}{2} \tau _q \right) d_j^* -\frac{1}{2} \tau _q^2 \, d_i^* \right) = 0, \end{aligned}$$

whence $\bar{N}_{ij} = 0$, i.e. $\bar{\boldsymbol{N}}$ is diagonal. Again,

32 $$\begin{aligned} \bar{N}_{ii} = \frac{\tau _q^2}{\rho \Theta _o (\tau _{q}+\tau _\theta )\Delta _i}, \quad i=1, 2, 3, \end{aligned}$$

with

33 $$\begin{aligned} \Delta _i = 1 + \frac{\tau _q \tau _{\upsilon }}{\tau _q + \tau _{\theta }} d_i^*. \end{aligned}$$

This tensor will be denoted $\boldsymbol{D}_N$ for convenience. Finally, tensor $\boldsymbol{N}$ can be obtained from $\bar{\boldsymbol{N}}$ by the transformation:

34 $$\begin{aligned} \boldsymbol{N} = S \bar{\boldsymbol{D}} S^T = \boldsymbol{N}^T. \end{aligned}$$

Tensor $\boldsymbol{G}$ is also diagonalized by *S* as follows:

$$\begin{aligned} \bar{\boldsymbol{G}}= & \frac{\tau _{\theta }}{\tau _q} \boldsymbol{D}_N \left[ \boldsymbol{I}_3 + \left( \tau _{\upsilon } + \frac{1}{2} \tau _q \right) \boldsymbol{D}^* \right] - \frac{1}{2} \tau _{\theta } \boldsymbol{D}^* \boldsymbol{D}_N, \\= & \frac{\tau _{\theta }}{\tau _q} \boldsymbol{D}_N \left( \boldsymbol{I}_3 + \tau _{\upsilon } \boldsymbol{D}^* \right) . \end{aligned}$$

Tensor $\boldsymbol{G}$ is obtained symmetric. For this tensor to be positive definite, it is sufficient that its eigenvalues be all non-negative:

35 $$\begin{aligned} \tau _{\upsilon } \le \frac{1}{\max \limits _{i} \vert d_i^* \vert }. \end{aligned}$$

In the end, the resulting dissipation function is non-negative.

The entropy and the heat capacity are obtained as:

36 $$\begin{aligned} \eta= & \eta _0 - \frac{1}{2} N'_{ij}Q_{i}Q_{j} - \frac{1}{2} M'_{ij} \left( Q_i \theta _{,j} + Q_j \theta _{,i} \right) - \frac{1}{2} P'_{ij} \theta _{,i }\theta _{,j} - \frac{1}{2} \mu '_{ij} \left( Q_i \upsilon _{,j} + Q_j \upsilon _{,i} \right) \nonumber \\ & \quad - \frac{1}{2} \beta '_{ij} \left( \theta _{,i} \upsilon _{,j} + \theta _{,j} \upsilon _{,i} \right) - \frac{1}{2} \alpha '_{ij}\upsilon _{,i} \upsilon _{,j}, \end{aligned}$$

37 $$\begin{aligned} c= & c_0 - \frac{1}{2} \theta \, N''_{ij}Q_{i}Q_{j} - \frac{1}{2} \theta \, M''_{ij} \left( Q_i \theta _{,j} + Q_j \theta _{,i} \right) - \frac{1}{2} \theta \, P''_{ij} \theta _{,i }\theta _{,j} - \frac{1}{2} \theta \, \mu ''_{ij} \left( Q_i \upsilon _{,j} + Q_j \upsilon _{,i} \right) \nonumber \\ & \quad - \frac{1}{2} \theta \, \beta ''_{ij} \left( \theta _{,i} \upsilon _{,j} + \theta _{,j} \upsilon _{,i} \right) - \frac{1}{2} \theta \, \alpha ''_{ij}\upsilon _{,i} \upsilon _{,j}, \end{aligned}$$

The expression for the heat capacity may be used, in conjunction with experimental data, to get information about the introduced material tensors.

One can easily show that the case of TPL reduces to that of DPL by taking the proper limits $\boldsymbol{K}^* \rightarrow \boldsymbol{0}$ and $\tau _{\upsilon } \rightarrow 0$, and that DPL reduces to the case of a single thermal relaxation time by taking $\tau _{\theta } \rightarrow 0$.

As in the case of DPL, when the thermal relaxation times, and tensors $\boldsymbol{K}$ and $\boldsymbol{K}^*$ are independent of temperature, the heat capacity keeps its equilibrium value.

## Simultaneous calculation of temperature and heat flux in dual-phase-lag

All the following considerations are restricted to the dual-phase-lag theory. According to what has been proposed above, the four unknowns {$\theta , Q_i$} satisfy the following set of partial differential equations: (i) the vector evolution equation for vector $\boldsymbol{Q}$ (equations (3) for DPL, (4) or (5) for TPL); (ii) the equation of energy, which can be expressed as follows for a rigid thermal conductor:

38 $$\begin{aligned} \rho \frac{\partial }{\partial t} \left( \psi + \Theta \eta \right) = - \text {div} \, \boldsymbol{Q}, \end{aligned}$$

with $\psi$ and $\eta$ given by their proper expressions in terms of $\theta$ and $Q_i$ and derivatives.

It is thus clear that obtaining a partial differential equation, whether linear or not, for the determination of temperature, independently of the heat flux, is not a simple matter, and may be achieved only in particular cases.

Nonlinear heat conduction has been treated by several authors due to its importance in practice. Nonlinearity may arise as a result of the temperature dependence of thermal conductivity and thermal relaxation times on temperature as in^29,32^. Presently, only the thermal conductivity is taken to vary linearly with temperature. However, the more complicated case when thermal relaxation times are variable as well is straightforward to deal with.

For the dual-phase-lag in a rigid thermal conductor, when the tensor of thermal conductivity is temperature dependent, but the relaxation times are constants, one has:

39 $$\begin{aligned} \psi= & - \frac{1}{2} \frac{c_0}{\Theta _0} \, \theta ^2 +\frac{1}{2} \frac{\tau _{q}^2}{\rho (\tau _{q} + \tau _{\theta })\Theta _o} K^{-1}_{ij}(\theta ) Q_{i}Q_{j} \nonumber \\ & \quad + \frac{\tau _q \tau _{\theta }}{\rho \Theta _0(\tau _{q} + \tau _{\theta })} Q_i \theta _{,i} + \frac{1}{2} \frac{\tau _{\theta }^2}{\rho (\tau _{q} + \tau _{\theta })\Theta _0} K_{ij}(\theta ) \theta _{,i }\theta _{,j}, \end{aligned}$$

40 $$\begin{aligned} \eta= & \frac{c_0}{\Theta _0} \, \theta - \frac{1}{2} \frac{\tau _{q}^2}{\rho (\tau _{q} + \tau _{\theta })\Theta _0} \, \frac{d}{d \theta } K^{-1}_{ij} \, Q_{i}Q_{j} \nonumber \\ & \quad - \frac{1}{2} \frac{\tau _{\theta }^2}{\rho (\tau _{q} + \tau _{\theta })\Theta _0} \, \frac{d}{d \theta }K_{ij}(\theta ) \, \theta _{,i }\theta _{,j}. \end{aligned}$$

We have taken

41 $$\begin{aligned} \psi _0 = - \frac{1}{2} \frac{c_0}{\Theta _0} \, \theta ^2, \end{aligned}$$

with $c_0$ a constant, hence

42 $$\begin{aligned} \eta _0 = - \frac{\partial \psi _0}{\partial \theta } = \frac{c_0}{\Theta _0} \, \theta . \end{aligned}$$

Then the equation of energy function is:

43 $$\begin{aligned} & \rho \frac{\partial }{\partial t}(\psi +\Theta \eta ) =\rho \frac{\partial }{\partial t}(\psi + (\theta +\Theta _0) \eta )= \nonumber \\ & \rho \frac{\partial }{\partial t} \left[ \frac{1}{2} \frac{c_0}{\Theta _0} \, \theta ^2 + c_0 \, \theta \right. \nonumber \\ & \qquad +\frac{1}{2} \frac{\tau _{q}^2}{\rho (\tau _{q} + \tau _{\theta })\Theta _o} \left( K^{-1}_{ij}(\theta ) - \Theta _0 \frac{d}{d \theta } K^{-1}_{ij}(\theta ) - \theta \frac{d}{d \theta } K^{-1}_{ij}(\theta ) \right) Q_{i}Q_{j} \nonumber \\ & \qquad + \frac{\tau _q \tau _{\theta }}{\rho \Theta _o(\tau _{q} + \tau _{\theta })} Q_i \theta _{,i} \nonumber \\ & \qquad \left. + \frac{1}{2} \frac{\tau _{\theta }^2}{\rho (\tau _{q} + \tau _{\theta })\Theta _o} \left( K_{ij}(\theta ) - \Theta _0 \frac{d}{d \theta } K_{ij}(\theta ) - \theta \frac{d}{d \theta } K_{ij}(\theta ) \right) \theta _{,i }\theta _{,j}\right] \nonumber \\ & \quad = - Q_{i,i}. \end{aligned}$$

In case the thermal conductivity tensor depends linearly on temperature, one has

$$\begin{aligned} K_{ij}=K^{(0)}_{ij}(1+\eta \theta )=K^{(0)}_{ij}+ K^{(0)}_{ij}\eta \theta \, , \end{aligned}$$

$$\begin{aligned} K^{-1}_{ij}=K^{(0)-1}_{ij}(1-\eta \theta )=K^{(0)-1}_{ij}-K^{(0)-1}_{ij}\eta \theta \, , \end{aligned}$$

and keeping only terms up to the second order of magnitude, Eq. (43) becomes:

44 $$\begin{aligned} & \frac{\rho c_0}{\Theta _0} \, \theta \dot{\theta } + \rho c_0 \, \dot{\theta } \nonumber \\ & \qquad + \frac{1}{2} \frac{\tau _{q}^2}{(\tau _{q} + \tau _{\theta })\Theta _0} \left( K^{(0)-1}_{ij} + \Theta _0 K^{(0)-1}_{ij}\eta \right) \left( Q_{i} \dot{Q_{j}} + Q_{j} \dot{Q_{i}} \right) \nonumber \\ & \qquad + \frac{\tau _q \tau _{\theta }}{\Theta _0(\tau _{q} + \tau _{\theta })} \left( Q_i \dot{\theta }_{,i} + \theta _i \dot{Q}_{,i} \right) \nonumber \\ & \qquad +\frac{1}{2} \frac{\tau _{\theta }^2}{(\tau _{q} + \tau _{\theta })\Theta _0} \left( K^{(0)}_{ij} - \Theta _0 K^{0}_{ij}\eta \right) \left( \dot{\theta _{,i }}\theta _{,j} + \theta _{,i }\dot{\theta _{,j}} \right) \nonumber \\ & \quad = - Q_{i,i}, \end{aligned}$$

The linear case may now be recovered as:

45 $$\begin{aligned} \rho c_0 \, \dot{\theta } = - Q_{i,i}. \end{aligned}$$

On the other hand, the evolution equation of heat flux in indicial form is:

46 $$\begin{aligned} Q_i+\tau _q \dot{Q_i} =- (K^{(0)}_{ij}+ K^{0}_{ij}\eta \, \theta ) \left( \theta _{,j} +\tau _{\theta } \dot{\theta _{,j }}\right) , \end{aligned}$$

## Application

The following application is intended to verify the reliability of the proposed model to deal with concrete problems of thermal wave propagation with finite speed. Consider the governing equations in a one-dimensional setting in a half-space $x \ge 0$ with the thermal conductivity *K* depending linearly on temperature. In contrast to^8^ in which a one-dimensional problem of heat propagation in a finite interval is considered, we have preferred to perform calculations in a half-space, in order to avoid complexity of the solution due to the existence of multiple reflections at both ends of the finite interval.

Identifying the characteristic scales for length, time, temperature and heat flux as (C.f.^10^):

47 $$\begin{aligned} L_0= \frac{\Theta _0 K^{(0)} }{Q_0}, \quad T_0 = \tau _0, \quad \Theta _0, \quad Q_0=\sqrt{\frac{\Theta _0^2 \rho c_0 K^{(0)}}{T_0}}, \end{aligned}$$

where $\tau _0$ is some charactersitic time that could be equal to $\tau _{q}$ or to $\tau _\theta$, but is left unspecifed for the time being, the nonlinear equations for the determination of $\theta$ and *Q* assume the final forms:

48 $$\begin{aligned} Q + \tau _{q} \frac{\partial Q}{\partial t}=-(1+\eta _0 \theta ) \left( \frac{\partial \theta }{\partial x} + \tau _\theta \frac{\partial ^2 \theta }{\partial x \partial t } \right) \end{aligned}$$

49 $$\begin{aligned} \begin{aligned}&\theta \frac{\partial \theta }{\partial t}+ \frac{\partial \theta }{\partial t} =-\frac{\partial Q}{\partial x} - \frac{{\tau _q} ^2 }{\tau _\theta +\tau _q } (1+ \eta _0) Q \frac{ \partial Q}{\partial t} \\&\quad - \frac{1}{2} \frac{\tau _\theta \tau _q }{\tau _\theta +\tau _q } \left( Q \frac{\partial ^2 \theta }{\partial t \partial x} +\frac{\partial Q}{\partial t}\frac{ \partial \theta }{\partial x} \right) - \frac{{\tau _\theta } ^2 }{\tau _\theta +\tau _q} (1 - \eta _0) \frac{\partial \theta }{\partial x}\frac{\partial ^2\theta }{\partial t \partial x } \end{aligned} \end{aligned}$$

Now write down system (48) and (49) in the symbolic matricial form:

50 $$\begin{aligned} \left( \begin{array}{ccc} \frac{\partial Q}{\partial t} \\ \\ \frac{\partial \theta }{\partial t} \end{array} \right) + \left( \begin{array}{ccc} 0 & \frac{1+ \eta _0 \theta }{\tau _q} \\ \\ \frac{1}{1 + \theta } & 0 \end{array} \right) \, \left( \begin{array}{ccc} \frac{\partial Q}{\partial x} \\ \\ \frac{\partial \theta }{\partial x} \end{array} \right) = \left( \begin{array}{ccc} f_1 \\ \\ f_2 \end{array} \right) , \end{aligned}$$

where small quadratic terms have been collected in $f_1$ and $f_2$, to find out that the $(2 \times 2)$ matrix multiplying the vector of spatial derivatives of the unknown functions has eigenvalues

$$\begin{aligned} \pm \sqrt{\frac{1+ \eta _0 \theta }{1 + \theta }} \, \frac{1}{\sqrt{\tau _q}}, \end{aligned}$$

which ensures that the system under consideration is of hyperbolic nature, and that the speed of propagation of the heat wave is approximately equal to the value $\frac{1}{\sqrt{\tau _q}}$.

### Initial and boundary conditions

The two nonlinear governing equations for temperature and heat flux (48) and (49) are solved numerically in the half-space under the following boundary and initial conditions in the time domain [0, 0.2]:

$$\begin{aligned} \theta (x,t)\Big |_{x,t=0} =0 , Q(x,t)\Big |_{x,t=0} =0 \end{aligned}$$

51 $$\begin{aligned} \theta (x,t)\Big |_{x_i,t} =1-\cos {(t)} , Q(x,t)\Big |_{x_i,t} =1-\cos {(t)} \end{aligned}$$

$$\begin{aligned} \theta (x,t)\Big |_{x_o,t} =0 , Q(x,t)\Big |_{x_o,t} =0 \end{aligned}$$

where $x_i=0$ and $x_o$ is taken sufficiently large for the radiation condition. The initial and boundary conditions have been taken in simple form so as to concentrate attention on the phenomenon of heat wave propagation (C.f.^16,29^). More involved conditions could be treated equally well.

### Numerical results and discussion

Let the parameters $\tau _{\theta }$, $\tau _{q}$, $\eta _0 = \eta \Theta _0$ assume the following numerical values for definiteness:

$$\begin{aligned} \tau _{\theta }=0.08,\quad \tau _{q}=0.5,\quad \eta _0=0.1 \, (\text { renamed } \eta \text { for the sake of brevity in what follows }) \end{aligned}$$

**Figure 1:**
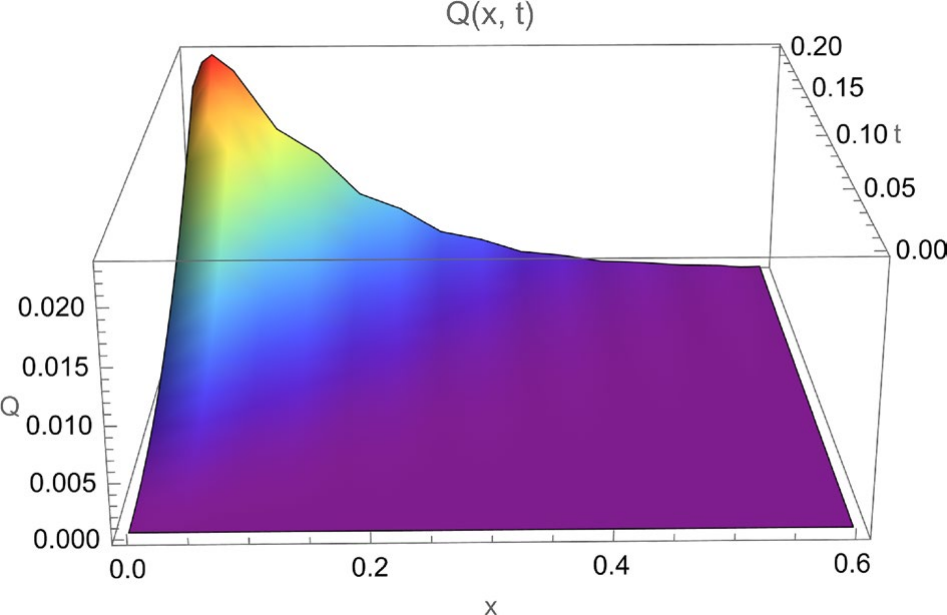
Heat flux *Q*(*x*, *t*) for $0 \le x \le 0.6$ and $0 \le t \le 0.2$.

**Figure 2:**
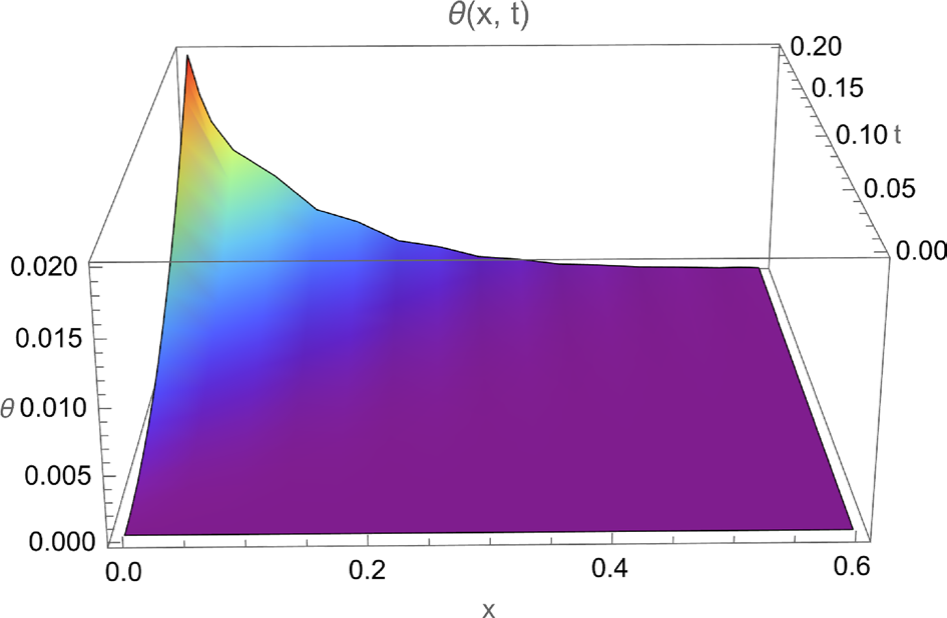
Temperature $\theta (x,t)$ for $0 \le x \le 0.6$ and $0 \le t \le 0.2$.

Figures 1 and 2 are 3D-plots of the heat flux and the temperature respectively. It is seen that these two functions tend to zero sufficiently far away from the boundary, as expected.

**Figure 3:**
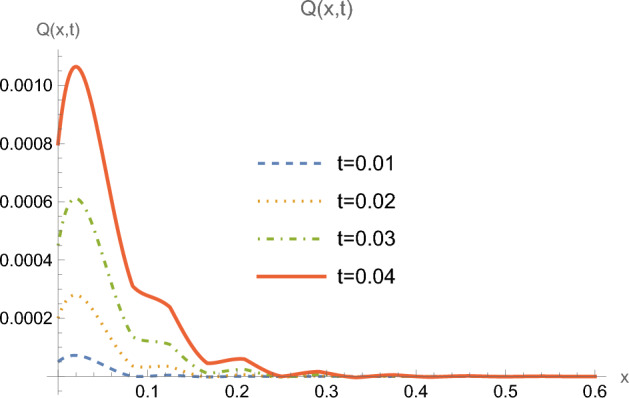
Heat flux *Q*(*x*, *t*) for $0 \le x \le 0.6$ and for multiple values of time *t*.

**Figure 4:**
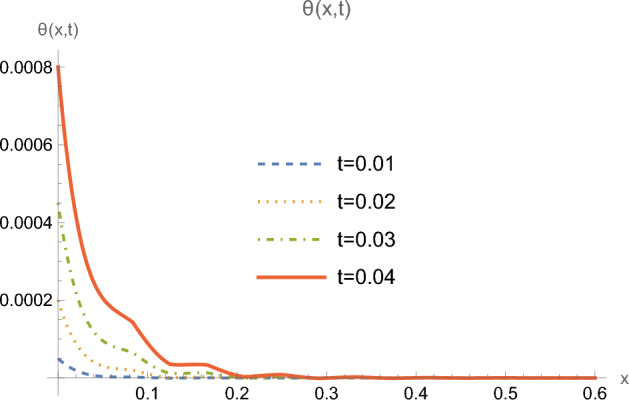
Temperature $\theta (x,t)$ for $0 \le x \le 0.6$ and for multiple values of time *t*.

Figures 3 and 4 illustrate the distributions of the heat flux and the temperature along the *x*-axis for many successive time moments. One clearly sees here the progression of the heat wave along the depth of the half-space with finite speed as time increases.

**Figure 5:**
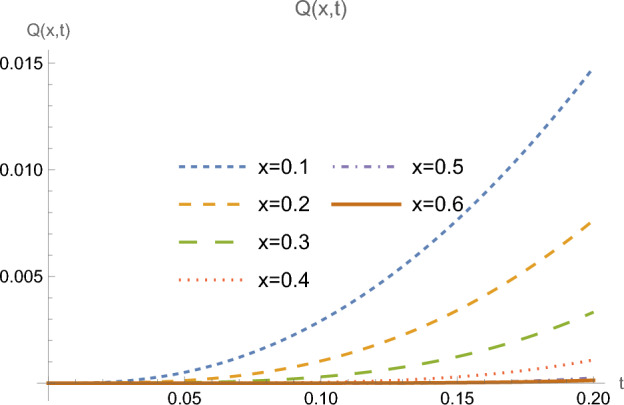
Heat flux *Q*(*x*, *t*) at multiple locations *x*.

**Figure 6:**
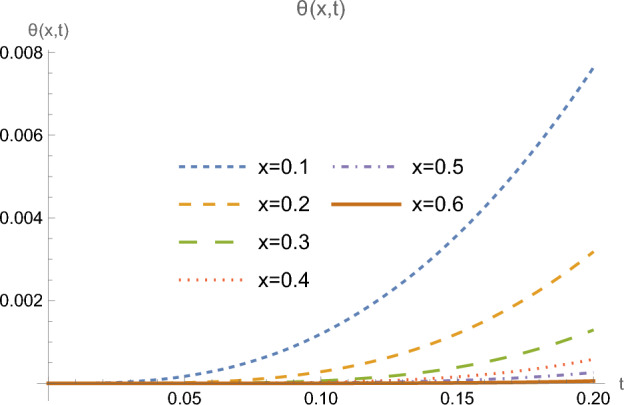
Temperature $\theta (x,t)$ at multiple locations *x*.

Figures 5 and 6 represent the distribution of heat flux and temperature as functions of time, for many successive locations in the half-space farther from the boundary. Here one clearly sees flattened initial portions of the curves along larger intervals, showing the retardation process in reaching the different locations, due to the finite speed of propagation of the heat wave.

**Figure 7:**
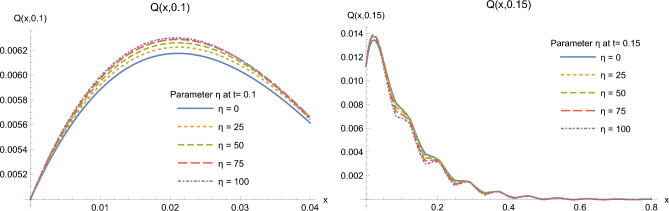
Heat flux *Q*(*x*, *t*) at times $t=0.1$ (left) and 0.15 (right) for some values of $\eta$.

**Figure 8:**
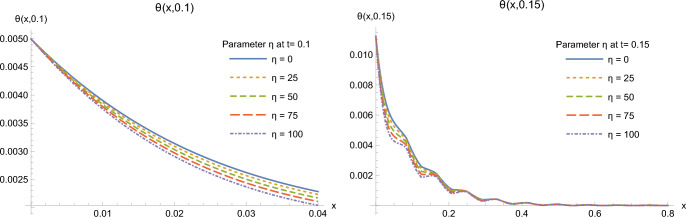
Temperature $\theta (x,t)$ at times $t=0.1$ (left) and 0.15 (right) for some values of $\eta$.

The effect of the nonlinearity parameter $\eta _0$, renamed $\eta$ for simplicity, on the solutions is illustrated on Figs. 7 and 8. Recall that this parameter stands for the dependence of heat conductivity on temperature. Here, we choose two time moments $t=0.10, 0.15$ to plot the solutions for different values of the nonlinearity parameter $\eta$ to illustrate its influence on the distributions of heat flux and temperature in the half-space. Two plots at different scales for each function allow to visualize conveniently the effect of parameter $\eta$. At time $t=0.10$, an increase of parameter $\eta$ seems to slightly increase the value of the heat flux and to slightly decrease the value of temperature. This picture is no more valid at $t=0.15$, so that one cannot formulate a clear conclusion concerning the effect of this nonlinearity parameter for all time moments.

## Conclusions

It has been shown that for concrete models of dual-phase-lag and three-phase lag in extended thermodynamics, and starting with well-known evolution equations for the heat flux, suitable simple forms of the free energy functions and corresponding dissipation functions can be constructed, that guarantee the non-negativeness of the dissipation function, hence the consistency of the models with the second law of thermodynamics. All the introduced material tensors are kept to relatively simple expressions with physical meaning, and may be calculated based on experimental data for the tensors of thermal conductivity, and the thermal relaxation times. The derived expressions for the entropy and the heat capacity contain additional terms, compared to their equilibrium values, which are quadratic forms in the heat flux, the gradient of temperature and the gradient of thermal displacement. The present work complements previous investigations (C.f.^19,20,35,36^) which use the classical expression for the dissipation function as the negative product of heat flux and temperature gradient. Moreover, it has the advantage of producing a free energy function and a dissipation function that reflect the independence of heat flux and temperature as thermodynamical arguments in the free energy for each of the two considered models. At all stages, the thermal conductivity is assumed to depend linearly on temperature, while the thermal relaxation times are taken constants.

Apart from the fact that all thermal relaxation times be positive, the only restriction imposed on them is expressed by inequality (35) concerning the phase lag for thermal displacement. Details concerning the effective values of the various thermal relaxation times to ensure stable solutions of the heat propagation problem may be found elsewhere (C.f.^18^).

The presently proposed model yields a system of four nonlinear partial differential equations, to be solved simultaneously for the determination of temperature and heat flux. A simplified version of these equations for DPL, when only quadratic nonlinearities are retained, is further investigated and solved numerically in a half-space to avoid complicated plots due to the appearance of multiply reflected waves. The obtained solution allows to illustrate the efficiency of the proposed model through 2D and 3D plots for the behavior of the solution, and to give a physical insight that points out at its adequacy in describing the propagation of finite speed heat waves in a rigid thermal conductor.

In the end, let us recall that the domain of validity of the presented work is that of Extended Irreversible Thermodynamics, the ability to propose generalized transport equations, free energies, dissipation functions and entropies, and to deal with the response of the thermodynamical system to high-frequency and short-wavelength perturbations, such as ultrasound propagation. These models may thus be important both for theory and for practical purposes in real situations where the system is out of equilibrium.

## Supplementary Information

Supplementary Information.

## Data Availability

All data generated or analyzed during this study are included in this article
