# Supplementary material for: Compatibility of the evolution equation for heat flux in dual-phase-lag and three-phase-lag with the principles of thermodynamics
Source: Sci Rep. 2026 Jan 20;16:2638. doi: 10.1038/s41598-025-33764-3 (PMC12824161; doi:10.1038/s41598-025-33764-3)
Supplement: Supplementary file 1 — Supplementary Information. [file 41598_2025_33764_MOESM1_ESM.pdf]

Compatibility of the evolution equation for heat flux in dual-phase-lag and three-phase-lag with the principles of thermodynamics

Asmaa Fawzy*, W. Mahmoud, E.K. Rawy, A.F. Ghaleb

Department of Mathematics, Faculty of Science, Cairo University, Giza, 12613, Egypt

*Corresponding author: Asmaa Fawzy email: asmaafawzy@gstd.sci.cu.edu.eg

Appendix

Simultaneous diagonalization of the positive-definite tensor $\boldsymbol{K}$ and the symmetric tensor $\boldsymbol{K}^*$.

Since $\boldsymbol{K}$ is positive definite and $\boldsymbol{K}^*$ is symmetric, and $\boldsymbol{K}\boldsymbol{K}^* = \boldsymbol{K}^*\boldsymbol{K}$, then one can diagonalize both tensors simultaneously. For this, let us diagonalize the symmetric tensor $\boldsymbol{K}^{-1/2}\boldsymbol{K}^*\boldsymbol{K}^{-1/2}$ using an orthogonal transformation O, say:

$$O^T\boldsymbol{K}^{-1/2}\boldsymbol{K}^*\boldsymbol{K}^{-1/2}O = D^* = \text{diag}\{d_1^*, d_2^*, d_3^*\}..$$

Take

$$S = K^{-1/2}O.$$

Hence:

$$\begin{aligned} S^T\boldsymbol{K}S &= O^TK^{-1/2}\boldsymbol{K}K^{-1/2}O = \boldsymbol{I}_3, \\ S^T\boldsymbol{K}^*S &= O^TK^{-1/2}\boldsymbol{K}^*K^{-1/2}O = D^*. \end{aligned}$$
